# Emergent severe acute respiratory distress syndrome caused by adenovirus type 55 in immunocompetent adults in 2013: a prospective observational study

**DOI:** 10.1186/s13054-014-0456-6

**Published:** 2014-08-12

**Authors:** Bing Sun, Hangyong He, Zheng Wang, Jiuxin Qu, Xuyan Li, Chengjun Ban, Jun Wan, Bin Cao, Zhaohui Tong, Chen Wang

**Affiliations:** Department of Respiratory and Critical Care Medicine, Beijing Chao-Yang Hospital, Capital Medical University, No. 8 Gongti Nanlu, Chaoyang district, Beijing, 100020 China; Beijing Institute of Respiratory Medicine, Beijing Key Laboratory of Respiratory and Pulmonary Circulation, Capital Medical University, No. 8 Gongti Nanlu, Chaoyang district, Beijing, 100020 China; Department of Infectious Diseases and Clinical Microbiology, Beijing Chao-Yang Hospital, Capital Medical University, No. 8 Gongti Nanlu, Chaoyang district, Beijing, 100020 China; Institute of Respiratory Medicine, Beijing Hospital, Ministry of Heath, No. 1 Dahua Road, Dongcheng district, Beijing, 100730 China

## Abstract

**Introduction:**

Since 2008, severe cases of emerging human adenovirus type 55 (HAdV-55) in immunocompetent adults have been reported sporadically in China. The clinical features and outcomes of the most critically ill patients with severe acute respiratory distress syndrome (ARDS) caused by HAdV-55 requiring invasive mechanical ventilation (IMV) and/or extracorporeal membrane oxygenation (ECMO) are lacking.

**Methods:**

We conducted a prospective, single-center observational study of pneumonia with ARDS in immunocompetent adults admitted to our respiratory ICU. We prospectively collected and analyzed clinical, laboratory, radiological characteristics, sequential tests of viral load in respiratory tract and blood, treatments and outcomes.

**Results:**

The results for a total of five consecutive patients with severe ARDS with confirmed HAdV-55 infection were included. All five patients were immunocompetent young men with a median age of 32 years. The mean time from onset to dyspnea was 5 days. Arterial blood gas analysis at ICU admission revealed profound hypoxia. Mean partial oxygen pressure/fraction of inspired oxygen was 58.1. Mean durations from onset to a single-lobe consolidation shown on chest X-rays (CXRs) and, from the first positive CXR to bilateral multilobar lung infiltrates, were 2 days and 4.8 days, respectively. The viral load was higher than 1 × 10^8^ copies in three patients and was 1 × 10^4^ in one patient. It was negative in the only patient who survived. The mean duration for noninvasive positive pressure ventilation (NPPV) failure and IMV failure were 30.8 hours and 6.2 days, respectively. Four patients received venovenous ECMO. Four (80%) of the five patients died despite receiving appropriate respiratory support.

**Conclusions:**

HAdV-55 may cause severe ARDS in immunocompetent young men. Persistent high fever, dyspnea and rapid progression to respiratory failure within 2 weeks, together with bilateral consolidations and infiltrates, are the most frequent clinical manifestations of HAdV-55-induced severe ARDS. Viral load monitoring may help predict disease severity and outcome. The NPPV and IMV failure rates were very high, but ECMO may still be the respiratory support therapy of choice.

**Trial registration:**

Clinicaltrials.gov NCT01585922. Registered 20 April 2012

## Introduction

Human adenoviruses (HAdVs) are notorious pathogens in people with compromised immune function and a frequent cause of outbreaks of acute respiratory disease among young children. Life-threatening adenoviral pneumonia has previously been documented among military trainees, patients with AIDS and transplant recipients [1–5]. Human adenovirus type 55 (HAdV-55), which is emerging as a highly virulent pathogen for acute fatal adenoviral pneumonia among immunocompetent adults in China, has gained increasing attention [6]. HAdV-55 is a newly identified, emergent acute respiratory disease pathogen causing two recent outbreaks in China in 2006 [7] and in Singapore in 2005 [8]. In 2011, this pathogen apparently re-emerged in Beijing, China, causing several cases of severe community-acquired pneumonia [9]. This pathogen was fully characterized by whole-genome sequencing [10]. Comparative studies showed that the ability of HAdV to cause severe disease may relate to the serotypes of HAdVs. Severe adenoviral pneumonia induced by HAdV-55 has been reported to be more closely related to severe cases compared to other serotypes (HAdV-3, HAdV-7 and HAdV-14) [6].

Current knowledge of HAdV-55-induced severe acute respiratory distress syndrome (ARDS) requiring invasive mechanical ventilation and/or extracorporeal membrane oxygenation (ECMO) support in immunocompetent adults is derived from single case reports or relatively small, single-center series. As a result, little information is available on HAdV-55 pneumonia complicated with severe ARDS, the frequency of which is expected to increase in the coming years. Here we describe the clinical features and outcomes of five prospective cases of HAdV-55 pneumonia complicated with severe ARDS in immunocompetent adults in our ICU.

## Material and methods

### Study population

Beginning in May 2012, a randomized trial of noninvasive positive pressure ventilation (NPPV) in ARDS patients was carried out in our center (ClinicalTrials.gov ID: NCT01585922). From May 2012 to April 2014, all adult patients with ARDS caused by pneumonia who were admitted to the respiratory ICU of Beijing Chao-Yang Hospital were prospectively enrolled. Severe ARDS was diagnosed according to the Berlin definition: (1) developing within 1 week of a known clinical insult or new or worsening respiratory symptoms; (2) bilateral opacities not fully explained by effusions, lobar and/or lung collapse, or nodules; (3) respiratory failure not fully explained by cardiac failure or fluid overload; (4) partial oxygen pressure/fraction of inspired oxygen (PaO_2_/FiO_2_) ≤100 mmHg with positive end-expiratory pressure (PEEP) ≥5 cmH_2_O; and (5) a chest radiograph with three or four quadrants with opacities. Patients with HAdV-55 infection and severe ARDS who failed conventional NPPV and invasive mechanical ventilation (IMV) were included in the analysis. This study was approved by the Institutional Review Board of Beijing Chao-Yang Hospital (LLKYPJ2012031). Data were analyzed anonymously. Each patient gave written informed consent for their data to be used for research and publication.

### Clinical data collection

Clinical information collected by investigators with a standardized data form included the following: demographic characteristics (age and sex), comorbidities, clinical symptoms (fever, cough, sputum, dyspnea, chest pain, rash, nausea, vomiting, abdominal pain, diarrhea and headache), signs (body temperature, heart rate, respiratory frequency, blood pressure and crackles in the lungs), laboratory tests (whole-blood cell count and blood chemistry) and microbiological findings and images of the lung (chest X-ray (CXR) and computed tomography). Concomitant medications, respiratory support, complications and outcomes were also recorded.

### Microbiological tests

Patients’ specimens, including sputum, whole blood and serum samples, were collected upon admission and during hospitalization. Microbiological tests were performed at the Department of Infectious Disease and Clinical Microbiology in our center, and the detection methods used were described in our previous report [6]. Common viruses causing respiratory illness were screened using a kit with 15 different viral assays. Serum samples were used for *Mycoplasma pneumoniae*, *Chlamydia pneumoniae* and *Legionella pneumophila* antibodies. All patients had their HAdV-55 infection confirmed by RT-PCR assay. Partial sequences of the hexon gene were analyzed to type the phylogeny of HAdV-55 strains. The adenoviral load was also performed on both respiratory specimens and blood by multiplex RT-PCR assay.

### Criteria for human adenoviral pneumonia

Viral pneumonia was diagnosed based on the presence of HAdV detected in sputum or throat swab samples by molecular methods.

### Statistical analysis

Continuous variables were summarized as mean ± standard deviation (SD) or median (interquartile range).

## Results

During the study period, a total of eight patients diagnosed with HAdV infection and respiratory failure were admitted to our ICU, and seven of them received a diagnosis of ARDS. Five consecutive patients with severe ARDS with confirmed HAdV-55 infection were admitted to our ICU between April and July 2013. They were included in the analysis. The other two patients had mild ARDS and were infected with other types of HAdVs.

### Demographics

All five patients were immunocompetent young men with a median age of 32 years (range, 28 to 40 years). All of the patients shared a B blood type and came from the same city: Baoding city, Hebei province, northern China. All patients had no exposure to farm animals, corn or hay. Patient 3 had tuberculosis pleuritis and received antituberculosis therapy at ICU admission. His blood tests, including the T-SPOT tuberculosis assay (Oxford Immunotec, Marlborough, MA, USA) and antibody of *Mycobacterium tuberculosis*, were negative.

### Clinical characteristics

Flulike symptoms, such as fever, cough and little sputum, were commonly observed at the onset of illness. All patients presented with a high fever, with a mean body temperature of 39.5°C (range, 39.0°C to 40.0°C), which persisted for 8 days (range, 6 to 11 days). Productive cough was observed in two patients. Dull substernal chest pain and rash were also observed in two patients. All patients had dyspnea. The mean time from onset to dyspnea was 5 days (range, 1 to 10 days). After the onset of dyspnea, patients usually progressed to respiratory failure or hypoxemia. The mean time from onset to ICU admission was 9.6 days (range, 8 to 11 days) (Table 1).

**Table 1:** **Demographic, clinical characteristics and laboratory values for immunocompetent adults with human adenovirus type 55 on the first day of admission** ^**a**^

**Patient**	**Length of onset to dyspnea (days)**	**Length of onset to ICU admission (days)**	**Length of NPPV (hours)**	**Length of IMV before ECMO (days)**	**Length of ICU stay (days)**	**Length of hospital stay (days)**	**T**_**max**_ **(°C)**	**P/F**	**Breathing rate (breaths/min)**	WBC (*10 ^**9**^ **/L)**	**AST (U/L)**	**Creatinine (μmol/L)**	**CK (U/L)**	**LDH (U/L)**	**HBDH (U/L)**
1	1	11	48	6	4	7	39	61.6	38	10	137	139.6	132	1345	845
2	10	11	24	4	5	9	39.5	62.5	40	5.17	190	78.5	1,653	1,666	953
3	8	10	36	6	20	20	39.4	60.9	40	1.83	264	75.6	4,892	1,313	751
4	2	8	22	2	19	24	40	49	52	2.1	213	70.2	2,586	1,577	1,079
5	4	8	24	13	16	16	39.6	56.5	45	4.69	168	85.5	89	1,363	938
Mean (±SD)	5 (3.9)	9.6 (1.5)	30.8 (11.1)	6.2 (3.7)	12.8 (7.7)	15.2 (7.2)	39.5 (0.4)	58.1 (5.6)	43 (6)	4.758 (3.3)	194.4 (48.0)	89.88 (28.3)	1,870.4 (1,992.8)	1,452.8 (158.2)	913.2 (123.1)

^a^AST, Aspartate aminotransferase (normal range = 10 to 42 U/L); CK, Creatine phosphokinase (normal range = 38 to 174 U/L); ECMO, Extracorporeal membrane oxygenation; HBDH, Hydroxybutyrate dehydrogenase (normal range = 72 to 182 U/L); IMV, Invasive mechanical ventilation; LDH, Lactate dehydrogenase (normal range = 85 to 250 U/L); NPPV, Noninvasive positive pressure ventilation; P/F: Partial oxygen pressure/fraction of inspired oxygen; T_max_, Maximum temperature; WBC, White blood cell count (normal range = 4.00 to 10.00*10^9^/L).

All patients had tachypnea when admitted to the ICU, with a mean rate of 43 breaths per minute (range = 38 to 52). Arterial blood gas analysis at ICU admission revealed profound hypoxia, with a mean PaO_2_/FiO_2_ of 58.1 (range = 49 to 62.5). White blood cell counts were low or in the normal range. All patients had elevated serum aspartate aminotransferase (AST), lactate dehydrogenase (LDH) and hydroxybutyrate dehydrogenase (HBDH) (Table 1). At admission, all patients’ levels of immunoglobulin (serum immunoglobulins G and M) and components C3 and C4 were in the normal range. Four patients had lower than normal T-cell subset counts (Table 2).

**Table 2:** **Humoral immunity and cell-mediated immunity on the immunocompetent adult infection with human adenovirus type 55** ^**a**^

**Patient**	**Blood type**	**T cells (** ***n*** **/ml)**	**T4 cells (** ***n*** **/ml)**	**T8-cells (** ***n*** **/ml)**	**IgG (mg/dl)**	**IgA (mg/dl)**	**IgM (mg/dl)**	**C3 (mg/dl)**	**C4 (mg/dl)**
Reference		(770 to 2,040)	(410 to 1,120)	(240 to 880)	(751 to 1,560)	(82 to 453)	(46 to 304)	(79 to 152)	(12 to 36)
1	B	317	200	107	858	142	163	102	20.4
2	B	NA	NA	NA	NA	NA	NA	NA	NA
3	B	599	347	225	1260	136	64.2	86.8	26.4
4	B	955	441	477	1640	110	92.3	38.7	13.1
5	B	599	347	225	1260	136	64.2	86.8	26.4

^a^Ig, Immunoglobulin; NA, Not available; T cells, CD3-positive cells; T4 cells, Both CD3- and CD4-positive cells; T8 cells, Both CD3- and CD8-positive cells.

### Radiographic features

**Figure 1:**
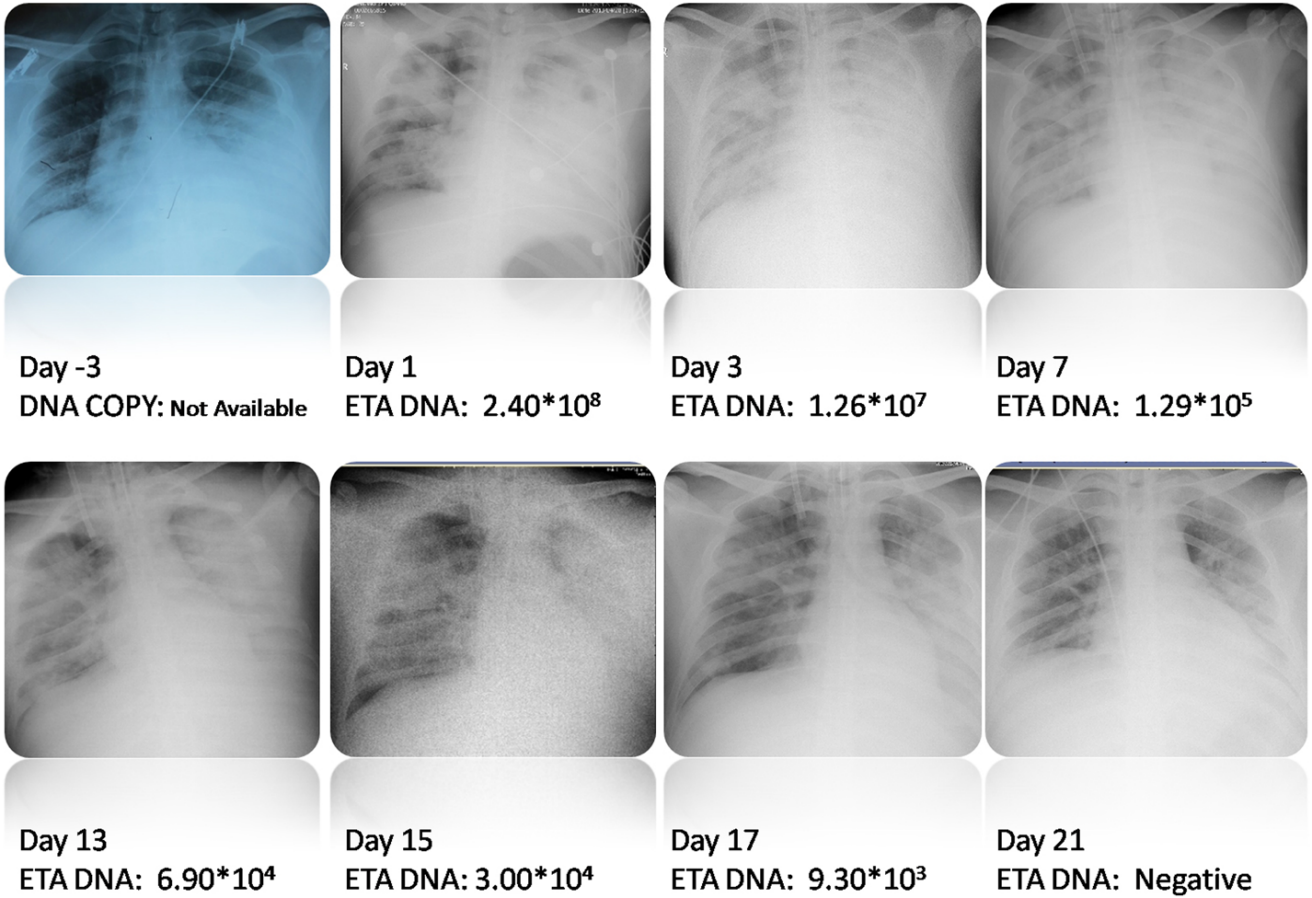
**Radiographic findings and their relationship to viral load monitoring in patient 4.** (Picture 1) Radiographs show widespread bilateral interstitial infiltrates at the onset of the disease. (Picture 2) The patient’s condition rapidly deteriorated within 3 days, and mechanical ventilation was required. (Picture 3) The patient’s adenoviral infection progressed, so supplementary oxygen was given on day 3 of admission. (Picture 4–8) Radiographs show gradual recovery with supportive therapy. Also shown are the trends of viral load detected with endotracheal aspirates (ETAs) and their relationship to radiological changes.

**Figure 2:**
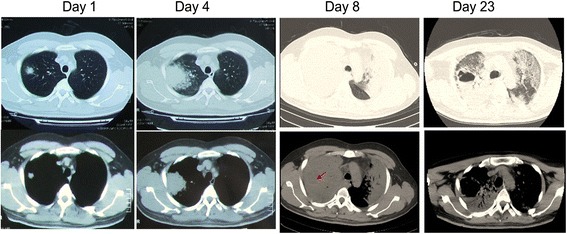
**Dynamic changes on computed tomography scans for human adenovirus type 55 pneumonia in patient 3.** Chest computed tomography scans taken on day 1 show a nodular shadow in the right upper lobe. The nodular shadow expanded dramatically within 3 days and was surrounded by ground-glass opacity on day 4. The lesion was diffuse in both lung fields on day 8. A cavity was observed on day 23, and the mediastinum window shows the formation of pulmonary abscess in the upper right lobe (red arrow). The lung abscess tested negative for *Legionella*, *Staphylococcus* and tuberculosis.

CXRs revealed multiple bilateral lobar or segment consolidation in the lungs of all five patients, and radiographic lesions progressed rapidly after ICU admission (Figure 1). Three patients were examined by high-resolution computed tomography (HRCT). Unilateral or bilateral consolidations and infiltrates were found on HRCT scans of all three of these patients. Consolidations within a single lobe or several lobes with a clear border and air bronchogram were the most common findings on HRCT scans. Nodules, patches, pleural effusion, abscess and a cavity were also seen visualized by HRCT (Figure 2). The mean duration from onset to a single-lobe consolidation on CXRs was 2 days (range = 1 to 5 days). The mean duration from the first positive CXR to bilaterally multilobar lung infiltrates was 4.8 days (range = 4 to 7 days).

### Detection of adenoviruses by RT-PCR

**Figure 3:**
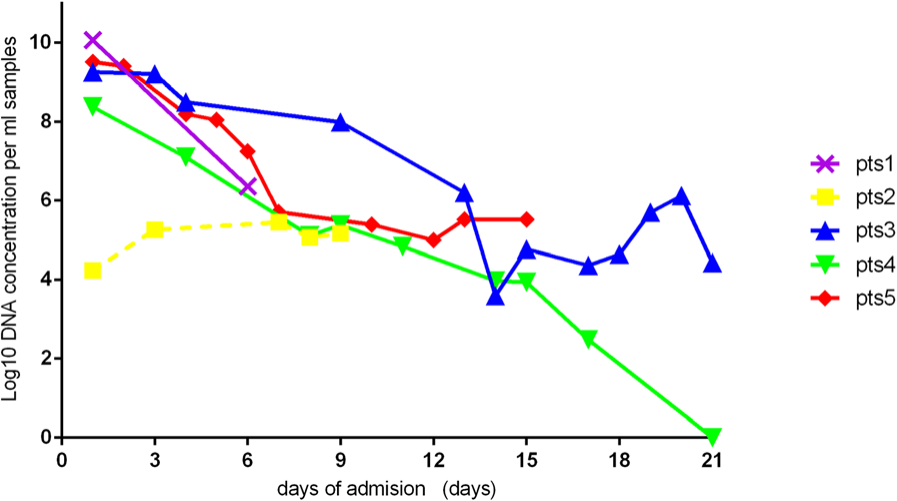
**Changes in human adenovirus type 55 concentration measured in endotracheal aspirates from all five acute respiratory distress syndrome patients.** Patient 4, whose viral DNA copies in ETA became negative, was the only patient who survived.

All patients had HAdV-55 viremia. In four of the five patients, it was first detected in endotracheal aspirate (ETA) samples. The time between initial ETA sample collection of adenoviruses and positive results for HAdV-55 nucleic acid in the blood was 1 to 10 days (Table 3). Virus DNA copies in ETAs were determined for all patients during their ICU stay. The viral load was higher than 1 × 10^8^ copies in three patients and 1 × 10^4^ in one patient. The viral load became negative in the only patient who survived. In the four patients who did not survive, DNA copies did not decrease, even with antiviral therapy (Figure 3).

**Table 3:** **Common respiratory pathogen coinfection with human adenovirus type 55 and patients’ clinical outcomes** ^**a**^

**Patient**	**Initial detection method**	**Days of positive HAdV in blood tests**	**Highest PCT level (ng/ml)**	**Bacteria**	**Fungus**	**G-test (pg/ml)**	**Outcome**	**Cause of death**
1	Sputum	2	5.66	Negative	Negative	<10	Death	ARDS
2	Throat swab	1	4.67	Negative	*Aspergillus fumigatus*	<10	Death	ARDS, IPA
3	Sputum	2	30.14	*Acinetobacter baumannii*, *Burkholderia cepacia*, *Staphylococcus*	Pneumocystis	164.7	Death	Septic shock, MODS
4	Sputum	10	1.58	*Acinetobacter baumannii*	Negative	<10	Survival	–
5	Sputum	2	0.48	*Acinetobacter baumannii*	Negative	256.1	Death	CRBSI

^a^ARDS, Acute respiratory distress syndrome; CRBSI, Catheter-related bloodstream infection; 1,3-beta-D-glucan, G-test (reference <20 pg/ml); HAdV, Human adenovirus; IPA, Invasive pulmonary aspergillosis; MODS, Multiple organ dysfunction syndrome; PCT, Procalcitonin (reference <0.05 ng/ml).

### Respiratory support

Oxygenation was not maintained with conventional NPPV or IMV support in any of the patients. The mean duration until NPPV failure was 30.8 hours (range = 22 to 48 hours), and the mean time until IMV failure was 6.2 days (range 2 = to 13 days) (Table 1). Four patients received venovenous ECMO to maintain oxygen saturation, and one patient refused ECMO support and received high-frequency oscillatory ventilation instead. Table 4 gives the oxygenation data of patients before and after venovenous ECMO support.

**Table 4:** **Oxygenation response to venovenous extracorporeal membrane oxygenation support** ^**a**^

**Patient**	**Conventional ventilation settings pre-ECMO**				**Pre-ECMO arterial blood gas analysis**				**Mechanical ventilation settings post-ECMO**				**Post-ECMO arterial blood gas analysis**			
	**Ventilator type**	**P**_**i**_ **/PS (cmH**_**2**_ **O)**	**PEEP (cmH**_**2**_ **O)**	**FiO**_**2**_ **(%)**	**pH**	**PaCO**_**2**_ **(mmHg)**	**PaO**_**2**_ **(mmHg)**	**SpO**_**2**_ **(%)**	**Ventilator type**	**PS (cmH**_**2**_ **O)**	**PEEP (cmH**_**2**_ **O)**	**FiO**_**2**_ **(%)**	**pH**	**PaCO**_**2**_ **(mmHg)**	**PaO**_**2**_ **(mmHg)**	**SpO**_**2**_ **(%)**
Patient 1: ECMO manufacturer/model (4,150 rpm; MAQUET (Wayne, NJ, USA)), 5.2 L/min, 6.8 L/min, total of 8 days on ECMO																
	PSV	12	20	1	7.229	62.3	76.7	65	PA/C	10	20	0.3	7.406	37.5	74.7	93
Patient 2: No ECMO, HFOV = 4 Hz, △P = 70 cmH_2_O, P_aw_ = 45 cmH_2_O																
	PA/C	18	10	0.8	7.267	81	76.3	94	No	–	–	–	–	–	–	–
Patient 3: ECMO manufacturer/model (3,950 rpm; MAQUET (Wayne, NJ, USA)), 5.0 L/min, 5.0 L/min, total of 6 days on ECMO																
	PA/C	12	20	1	7.25	66.4	75.4	90	PA/C	10	18	0.4	7.517	41.2	71.5	96
	HFOV = 4.5 Hz, △P = 65 cmH_2_O, P_aw_ = 26 cmH_2_O															
Patient 4: ECMO manufacturer/model (3,740 rpm; MAQUET (Wayne, NJ, USA)), 4.38 L/min, 4.0 L/min, total of 2 days on ECMO																
	SIMV	24	10	1	7.44	38	44	76	PA/C	10	14	0.35	7.441	37.5	89.6	96.8
Patient 5: ECMO manufacturer/model (3,340 rpm; MAQUET (Wayne, NJ, USA)), 4.9 L/min, 4.5 L/min, total of 13 days on ECMO																
	PA/C	16	18	1	7.347	74.2	80.2	83	PA/C	12	14	0.3	7.485	40.8	103	96
	HFOV = 4 Hz, △P = 65 cmH_2_O, P_aw_ = 30 cmH_2_O															

^a^ECMO, Extracorporeal membrane oxygenation; FiO_2_, Fraction of inspired oxygen; HFOV, High-frequency oscillatory ventilation; PA/C, Pressure assist/control; PaCO_2_, Partial pressure of carbon dioxide; PaO_2_, Partial pressure of oxygen; P_aw_, Airway pressure; PEEP, Positive end-expiratory pressure; P_i_, Initial pressure; PS, Sustained pressure; PSV, Pressure support ventilation; SIMV, Synchronized intermittent mechanical ventilation; SpO_2_, Peripheral capillary oxygen saturation; rpm, rounds per minute. The two liters per minute means the range of blood flow provided at the pump rounds level of ECMO.

### Antimicrobiological therapy and outcome

All patients received antiviral therapy, including acyclovir (10 mg/kg, every 8 hours, intravenous drip), ganciclovir (5 mg/kg, every 12 hours, intravenous drip) and ribavirin (250 mg, twice daily, intravenous drip). Considering that bacterial coinfection may combine with a severe viral infection, broad-spectrum intravenous antibiotics were given to all patients. Tests for bacterial pathogens were negative for only one patient (Table 3). Four (80%) of the five patients died. Among the four patients receiving venovenous ECMO, only one patient survived. The other four patients died due to ARDS, *Aspergillus fumigatus* coinfection, septic shock and catheter-related bloodstream infection due to *Acinetobacter baumannii*, respectively*.*

## Discussion

To the best of our knowledge, this is the first cohort observational study on the clinical characteristics of patients with severe ARDS caused by emergent HAdV-55 infection and also the first on the evaluation of a viral load test for monitoring the reaction to therapy and for prediction of patient outcome. The following are the main findings of this study. (1) HAdV-55 may cause severe ARDS in immunocompetent young men with blood type B. All of our patients were from the same city of Hebei province, northern China. (2) Persistent high fever, dyspnea and rapid progression to respiratory failure within 2 weeks, together with bilateral consolidations and infiltrates at the same time, are the most frequent clinical manifestations of severe HAdV-55-induced ARDS. (3) Viral load monitoring may help predict disease severity and patient outcome. (4) The NPPV and IMV failure rates were very high, and ECMO may be the last support method for this group of patients. (5) HAdV-55-induced severe ARDS has a very high mortality rate (80%) despite appropriate respiratory support.

Sporadic severe adenoviral infection in healthy adults has historically been described for serotype 4 [11], serotype 7 [4,12] and, more recently, serotype 14 in the general population and in military trainees [13,14]. HAdV-55 was first completely characterized in Shaanxi, China [7] and then reemerged in Hebei, a province close to Beijing, where it caused several cases of acute respiratory disease [9]. It was presumed that HAdV-55 was a recombinant form of the B2 species of HAdV-14 and HAdV-11 [7,15] due to its sharing a hexon gene with the HAdV-11 and HAdV-14 chassis [16]. The results of our study show that HAdV-55, as an emerging pathogen among immunocompetent adults, may cause severe ARDS.

The prevalence of severe fatal adenoviral pneumonia induced by HAdV-55 in our study is somewhat similar to that described by Cao and colleagues [6]. All cases of reported HAdV-55 in our study were from the same city: Baoding, Hebei province, northern China. They occurred between April and July 2013, just partly overlapping or following the influenza epidemic. The patients with severe disease also came from the same region and were treated during a similar time period, which suggests that HAdV-55 may be an important viral pathogen derived from this region.

Our study results suggest that the following may be clinical features of ARDS caused by HAdV-55: persistent high fever, rapid progression of dyspnea, need for mechanical ventilation support, elevated AST level and rapid progression from unilateral infiltrates to bilateral consolidations. These clinical features are highly similar to those of ARDS caused by other types of HAdV described in previous reports [6,9].

Recent studies have shown that the immune system plays a crucial role in the clearance of HAdV viremia and survival of the host [17]. Chen *et al*. reported that, in the acute phase of HAdV-55 infection, patients with severe disease may have high levels of dendritic cells and Th17 cells [18]. In our study, the only patient who recovered from severe infection had higher T-cell counts. Three of the five patients had relatively low T-cell counts when admitted. Our results suggest that these three patients may have been relatively immunocompromised and that a lower T-cell count may be a risk factor for HAdV-55 infection in young adults.

HAdV-55 DNA was previously reported in 41.2% of patients with severe infection [18]. In our study, HAdV-55 DNA was detected and monitored in all patients with severe ARDS. The initial, and trend of, viral load that presented as HAdV-55 DNA copies in the respiratory tract samples and blood may suggest the severity of infection and may predict both the reaction to therapy and patient outcome.

The use of mechanical ventilation and ECMO in patients with ARDS caused by HAdV-55 has not been detailed in previous studies. In our cohort, we found that severe HAdV-55 infection could cause a rapid progression of respiratory failure, with a very high failure rate for NPPV and IMV. This failure rate may be a result of the large area of consolidation that induced a severe shunt in the lung, which may lead to lack of response to positive pressure ventilation. For patients with severe ARDS, ECMO should be considered a better choice for oxygenation.

Our study has limitations. It is an observational study with no comparison group, so the difference between the severe and modest infections could not be clarified in terms of immune status, clinical features, radiological findings, viral load and treatment effects on respiratory support and antiviral therapy. Sequential dynamic analysis is needed to determine the relationship between HAdV-55 viremia and treatment response.

## Conclusions

Our data provide new insight into the clinical features of HAdV-55 infection in patients with severe ARDS. HAdV-55 may cause severe ARDS in immunocompetent young men. Persistent high fever, dyspnea and rapid progression to respiratory failure within 2 weeks, together with bilateral consolidations and infiltrates during the same period, are the most frequent clinical manifestations of severe HAdV-55-induced ARDS. Viral load monitoring may help predict disease severity and patient outcome. NPPV and IMV failure rates were very high, and thus ECMO may be a better choice of respiratory support in this group of patients. HAdV-55-induced severe ARDS has a very high mortality rate (80%) despite appropriate respiratory support.

## Key messages

- HAdV-55 infection may be a cause of severe ARDS in immunocompetent young male adults.
- Persistent high fever, dyspnea, rapid progression to respiratory failure within 2 weeks, together with bilateral consolidations and infiltrates during the same period, are the most frequent clinical manifestations of severe HAdV-55-induced ARDS.
- Viral load monitoring may be helpful in predicting patients’ disease severity and outcome.
- NPPV and IMV failure rates were very high, and thus ECMO may be a better choice for respiratory support in this group of patients.
- HAdV-55-induced severe ARDS has a very high mortality rate of 80% despite appropriate respiratory support.

## Abbreviations

ARDS: Acute respiratory distress syndrome
CXR: Chest X-ray
ECMO: Extracorporeal membrane oxygenation
ETA: Endotracheal aspirate
HAdV-55: Human adenovirus type 55
IMV: Invasive mechanical ventilation
NPPV: Noninvasive positive pressure ventilation
PEEP: Positive end-expiratory pressure
